# Using the maternal-fetal genotype incompatibility test to assess non-inherited maternal HLA-DRB1 antigen coding alleles as rheumatoid arthritis risk factors

**DOI:** 10.1186/1753-6561-1-s1-s124

**Published:** 2007-12-18

**Authors:** Hsin-Ju Hsieh, Christina GS Palmer, Sinead Harney, Hsiu-Wen Chen, Lara Bauman, Matthew A Brown, Janet S Sinsheimer

**Affiliations:** 1 Genentech, Inc., 1 DNA Way, South San Francisco, California 94080, USA; 2 Departments of Psychiatry and Biobehavioral Sciences, University of California, 695 C.E. Young Drive South, Los Angeles, California 90095, USA; 3 Human Genetics, University of California, 695 C.E. Young Drive South, Los Angeles, California 90095, USA; 4 Department of Rheumatology, Cork University Hospital, College Road, Wilton, Cork, Ireland; 5 Biostatistics, University of California, 695 C.E. Young Drive South, Los Angeles, California 90095, USA; 6 Biomathematics, University of California, 695 C.E. Young Drive South, Los Angeles, California 90095, USA; 7 Centre for Immunology and Cancer Research, University of Queensland, Princess Alexandra Hospital, Woolloongabba, Queensland 4102, Australia

## Abstract

Non-inherited maternal antigens encoded by specific *HLA-DRB1 *alleles (NIMA) have been implicated as a rheumatoid arthritis (RA) risk factor. Using genotype data from North American Rheumatoid Arthritis Consortium study participants and the maternal-fetal genotype incompatibility (MFG) test, we find evidence for offspring allelic effects but no evidence for NIMA as a RA risk factor. We discuss possible reasons why our result conflicts with several previous studies (including one of our own) that used RA patients from northern Europe.

## Background

Rheumatoid arthritis (RA) is highly associated with HLA-DRB1 *0101, *0102, *0401, *0404, *0405, *0408, *0423, *10, *1001, *1402, and *1406 alleles that encode a shared epitope (SE) [1]. However, as many as 30% of the patients do *not *carry SE coding alleles [1] and an association between non-inherited maternal SE *HLA-DRB1 *antigen coding alleles (NIMA) and RA also has been observed [2-5]. One explanation for the latter finding is that NIMA may be involved in RA pathogenesis through microchimera formation in offspring who do not have SE coding alleles [2]. However, other studies have not found a significant NIMA effect [6-8].

Hsieh et al. [3] demonstrated that the maternal-fetal genotype incompatibility (MFG) test [9,10] allows the joint estimation of the offspring allelic and NIMA effects as a RA risk factor and is robust to population stratification. The current study applies the MFG test to *HLA-DRB1 *genotype data from the North American Rheumatoid Arthritis Consortium (NARAC) study to test NIMA as a risk factor for RA.

## Methods

### Statistical modeling: the MFG test

The MFG test models the joint distribution of parental and offspring genotypes given the number of affected offspring in nuclear families as

$$\prod_{i=1}^{N}\mathrm{Pr}(G_{c}^{\left(i\right)},G_{p}^{\left(i\right)}|D^{\left(i\right)}),$$

where for family *i *= 1,..., *N*, $G_{c}^{\left(i\right)}=\left(G_{c1}^{\left(i\right)},\cdots ,G_{cn_{i}}^{\left(i\right)}\right)$ denote the genotypes of the *n*_*i *_offspring, in which *k*_*i *_are affected; $G_{p}^{\left(i\right)}=\left(G_{m}^{\left(i\right)},G_{f}^{\left(i\right)}\right)$ denote parental genotypes, in which $G_{m}^{\left(i\right)}$ and $G_{f}^{\left(i\right)}$ are maternal and paternal genotypes, respectively; $D^{\left(i\right)}=\left(D_{1}^{\left(i\right)},\cdots ,D_{k_{i}}^{\left(i\right)}\right)$ denotes the event that *k*_*i *_offspring are affected [4,5]. Phenotypes of the unaffected offspring are not used in the likelihood.

Using Bayes' theorem and assuming siblings' phenotypes are independent given parental genotypes, likelihood Eq. (1) is parameterized in terms of penetrance functions and population mating type frequencies [3,9]. As in Hsieh et al. [3], we model the penetrances as

pr(*D*|*G*_*c*_, ***G***_*p*_) = *p *× *μ*^*I*[*M*] ^× *ρ*_1_^*I*[*Z *= 1] ^× *ρ*_2_^*I*[*Z *= 2] ^,

where *I *[·] is the indicator function, *M *is the event that the offspring does not inherit an SE coding allele carried by the mother (i.e., NIMA), and *Z *denotes the number of SE coding alleles present in the offspring genotype. The parameter *p *denotes the population baseline disease incidence rate, which ultimately cancels from Eq. (1). The parameter *μ *is the relative risk due to NIMA; *ρ*_1 _and *ρ*_2 _are the relative risks when one or two copies of the SE coding allele are present in the offspring genotype, respectively, relative to zero copies. Note that the relative risks (*μ*, *ρ*_1_, *ρ*_2_) range from 0 to infinity, and each have a null value of 1. The numerical maximization of the log-likelihood is better conditioned when we use the natural logarithm of the relative risks as parameters so we actually estimate *α *= log(*μ*), *β*_1 _= log(*ρ*_1_), and *β*_2 _= log(*ρ*_2_) [9]. Each of these parameters, *α*, *β*_1_, and *β*_2_, are therefore defined on the real line from negative infinity to infinity and each have a null value of zero.

Because we assume that all SE coding alleles confer the same risk to RA susceptibility (as was assumed in earlier studies [2-8]), the model reduces to two alleles that we denote as *S *for the SE coding risk allele and *N *for the non SE coding allele. Thus, there are three possible genotypes (*S*/*S*, *S*/*N*, and *N*/*N*). The penetrance function as expressed by Eq. (2) is short-hand for three mutually exclusive maternal-offspring genotype combinations in which the indicator functions determine which relative risk corresponds to the genotype combination. These mutually exclusive combinations are: the offspring carries two copies of the S allele; the offspring carries one copy of the S allele; and the offspring does not carry S allele but the mother does. Each of these combinations has an associated risk that we define in a standard manner, that is, as relative to a reference category in which both the offspring and mother are *N*/*N*.

Assuming mating symmetry under the null hypothesis, there are six possible mating types [10]. Neither Hardy-Weinberg equilibrium nor random mating is assumed. Data from families with incomplete parental genotypes are included in Eq. (1) by assuming the genotypes are missing at random and summing over all possible parental genotypes [9,11]. Hypothesis tests of offspring allelic or of NIMA effects use a likelihood-ratio test statistic, whose distribution is asymptotically chi-square.

### The NARAC data set

Of the four GAW15 RA study samples, only the NARAC data set was suitable for this analysis because at least some parental *HLA-DRB1 *genotypes must be available to test for NIMA effects. The ethnic background of the NARAC families is representative of United States and Canadian families affected with RA (predominately Caucasian with African, Hispanic, Native American, and Asian minorities).

Before recoding the alleles into *S *and *N*, we used the pedigree trimming option of Mendel (version 6.5.0) to remove individuals without *HLA-DRB1 *genotypes who are not necessary to define the relationships among genotyped individuals [12,13]. We then tested for genotyping errors using the mistyping analysis available in Mendel version 6.5.0 [13,14] and found that the *HLA-DRB1 *error rate was less than 1%. Individuals' genotypes that were in error were omitted and when several family members could be in error, the entire family's *HLA-DRB1 *genotypes were omitted. Most of the families are nuclear families; however there are a few extended families. For each extended pedigree, a single nuclear family was selected using the following protocol: whenever a nuclear family has complete parental genotypes available, they are selected over those families with zero or one parent genotyped. Whenever multiple nuclear families within the extended pedigree have the same parental genotype availability, one of the nuclear families is randomly selected. At this point, there were 708 nuclear families.

About half the families have neither parent genotyped. These families provide very little power for detecting maternal-fetal genotype interactions [11] and were omitted. The remaining number of nuclear families was 318. We then recoded the alleles. The resolution of 119 individuals' *HLA-DRB1 *genotypes is too low to allow certain classification of their alleles as *S *or *N*. For example the allele *01 could be *0101 (*S*) or *0103 (*N*). These ambiguous genotypes were treated as missing in the analysis.

The final data set had 263 nuclear families with at least one parent and one affected offspring genotyped. The distribution of affected offspring and genotyped parents is given in Table 1. There are a total of 517 affected offspring (393 female and 124 male) in these families. To test whether we introduced a bias by our family selection scheme, we compared the genotype distribution of affected offspring from the families used in the analysis to the genotype distribution of affected offspring from the excluded families. Specifically, we randomly selected one affected offspring per included family and one affected offspring per excluded family and compared the distributions using a Fisher exact test. We found that the genotype distributions were not significantly different (*p *= 0.946).

**Table 1:** Distribution of affected offspring among the 263 families

No. affected siblings	Families with both parents genotyped	Families with only mothers genotyped	Families with only fathers genotyped
1	6	18	6
2	53	135	25
3	4	12	3
4	0	1	0

## Results

### Inference

We fit three models by placing different constraints on the parameters. These models are compared by constructing likelihood-ratio test statistics. Comparison of Models 1 (*α *= *β*_1 _= *β*_2 _= 0) and 2 (*α *= 0) in Table 2 reveals a highly significant offspring SE allelic effect on RA risk ($\chi_{2}^{2}$ = 128.1, *p *= 1.5 × 10^-28^). The relative risk for individuals with S/N is *ρ*_1 _= 4.3 and the relative risk for individuals with S/S is *ρ*_2 _= 12.7, similar in magnitude to the results of earlier association studies (see for example [15,16]). Comparison of Models 2 (*α *= 0) and 3 (no parameter restrictions) shows there is no evidence for the NIMA effect on RA risk in the presence of offspring allelic effects ($\chi_{1}^{2}$ = 0.0172, *p *= 0.8956).

**Table 2:** Offspring shared epitope and NIMA effects as estimated by the MFG test

Model	*α *= log*μ *(std err)	*β*_1 _= log*ρ*_1 _(std err)	*β*_2 _= log*ρ*_2 _(std err)	log likelihood
1	0	0	0	-731.0926
2	0	1.462(0.188)	2.538(0.239)	-667.0471
3	-0.0423 (0.324)	1.436 (0.272)	2.511 (0.317)	-667.0385

### Power

One possible explanation for our failure to find evidence for the NIMA effect is that our sample size is too small. The earlier studies found the MFG effect to be ~4, approximately equal to the S/N risk to offspring [2-5]. We simulated 1000 data sets and found that there is 80% power to detect an MFG effect size of 3.5 with 63 families each having two affected offspring and completely genotyped parents. Our actual study sample has power to detect even smaller effect sizes because the additional 200 families with only one parent genotyped substantially increase the power [11].

## Discussion

MFG incompatibility results when specific maternal-fetal genotype combinations produce an adverse effect on the developing fetus that ultimately increases offspring disease risk. The exposure of fetuses who do not carry SE coding alleles to a maternal SE antigen is an example of a putative MFG incompatibility event and so can be examined using the MFG test.

The highly significant offspring allelic effects are consistent with numerous studies [3,15,16]. However, we did not find any evidence of a NIMA effect. Although we can not exclude the possibility that small NIMA effects were missed, low power is not a reason for the failure to detect moderate NIMA effects. Our simulation results show that we could detect a NIMA effect that is less than the effect size observed in earlier studies [2-5]. Although the NARAC families are ethnically diverse, population stratification also is not a concern as the MFG test is robust to its effects [3,10,11].

There were originally 708 nuclear families available in the NARAC data set. After excluding families without parental genotypes and individuals with low-resolution *HLA-DRB1 *genotypes, only 263 nuclear families were used in our analysis. However when the genotypes are missing at random, it has been shown that MFG test produces unbiased parameter estimates and accurate hypothesis tests when only approximately 25% of the families in the sample have both parental genotypes [11]. The missing-at-random assumption is reasonable in this study. Therefore, the remaining 263 families provide valid, unbiased results even though more than half of the original NARAC families are excluded.

The following reasons might account for the failure to find a significant MFG incompatibility effect: a) the previous studies with positive findings [2-5] could represent type I errors, or the maternal or offspring effects at other *HLA *loci could have been misattributed to a *HLA-DRB1 *NIMA effect (an issue of model misspecification); b) *HLA-DRB1 *is difficult to accurately genotype and mistyping could lead to both false-positive and negative NIMA results; c) there could be population differences in the susceptibility to microchimera; d) differences in ascertainment or diagnostic criteria used in the different studies might also contribute to the variability in the results. The NARAC families are multi-case families and these families may have a different genetic background than single-case families [5].

## Conclusion

The MFG test [3,9-11] can be used to determine whether the *HLA-DRB1 *NIMA effect is a risk factor for RA. Although we can not rule out NIMA as a RA risk factor of small effect in the NARAC families, we find no evidence for NIMA as a RA risk factor of moderate effect.

## Competing interests

The author(s) declare that they have no competing interests.
